# Characterization of the layer, direction and time-dependent mechanical behaviour of the human oesophagus and the effects of formalin preservation

**DOI:** 10.1098/rsif.2023.0592

**Published:** 2024-04-10

**Authors:** Ciara Durcan, Mokarram Hossain, Grégory Chagnon, Djordje Perić, Edouard Girard

**Affiliations:** ^1^ Zienkiewicz Institute for Modelling, Data and Artificial Intelligence, Faculty of Science and Engineering, Swansea University, Swansea SA1 8EN, UK; ^2^ CNRS, UMR 5525, VetAgro Sup, Grenoble INP, TIMC, Grenoble Alpes University, Grenoble 38000, France; ^3^ Laboratoire d’Anatomie des Alpes Françaises, Grenoble Alpes University, Grenoble, France

**Keywords:** gastrointestinal tract, biomechanics, uniaxial tensile testing, zero-stress state, cadaver preservation

## Abstract

The mechanical characterization of the oesophagus is essential for applications such as medical device design, surgical simulations and tissue engineering, as well as for investigating the organ’s pathophysiology. However, the material response of the oesophagus has not been established *ex vivo* in regard to the more complex aspects of its mechanical behaviour using fresh, human tissue: as of yet, in the literature, only the hyperelastic response of the intact wall has been studied. Therefore, in this study, the layer-dependent, anisotropic, visco-hyperelastic behaviour of the human oesophagus was investigated through various mechanical tests. For this, cyclic tests, with increasing stretch levels, were conducted on the layers of the human oesophagus in the longitudinal and circumferential directions and at two different strain rates. Additionally, stress-relaxation tests on the oesophageal layers were carried out in both directions. Overall, the results show discrete properties in each layer and direction, highlighting the importance of treating the oesophagus as a multi-layered composite material with direction-dependent behaviour. Previously, the authors conducted layer-dependent cyclic experimentation on formalin-embalmed human oesophagi. A comparison between the fresh and embalmed tissue response was carried out and revealed surprising similarities in terms of anisotropy, strain-rate dependency, stress-softening and hysteresis, with the main difference between the two preservation states being the magnitude of these properties. As formalin fixation is known to notably affect the formation of cross-links between the collagen of biological materials, the differences may reveal the influence of cross-links on the mechanical behaviour of soft tissues.

## 1. Introduction

The oesophagus is the organ of the upper digestive tract that transports food and drink, collectively known as the fluid bolus, from the pharynx to the stomach via a relatively simple but highly effective mechanical process. This process, called peristalsis, is responsible for the transport of various matters throughout the body [1–3] and consists of wave-like contractions of the hollow organs’ muscular wall. For the oesophagus, its primary role of bolus transport is a result of a combination of the passive and active properties of the tissue wall [4,5], as well as the interaction of the forces generated with the hydrodynamic fluid bolus [6]. Therefore, it is crucial to understand each of these aspects to assess how they contribute to the function of the oesophagus in both health and disease. Out of these aspects, the passive properties may be viewed as fundamental, providing the baseline properties that allow transport to take place [4]. Notably, the oesophagus is made of two main composite layers, the muscularis propria (muscular layer) and the mucosa-submucosa (mucosal layer), which are thought to individually contribute to the organ’s passive material response [7].

There have been a number of *in vivo* studies performed on the human oesophagus [8–16], including those that looked at its passive properties by administrating muscle relaxants, such as atropine and butylscopolamine, to the volunteers in the study [13,16]. To the best of the authors’ knowledge, Frøkjær *et al.* [15,16] were the only ones to study the direction-dependent and layer-dependent behaviour of the human oesophagus. They found, through their *in vivo* tests on human subjects, that distension resulted in tension in the circumferential direction, shortening in the longitudinal direction and compression in the radial direction [16]. Furthermore, they found that material stiffness was lowest at the mucosal (inner) surface and increased throughout the oesophageal wall, while circumferential stress and strain decreased through the wall and were lowest at the outer (muscular) surface [16]. However, as the mucosal layer is folded *in vivo*, Frøkjær *et al.* [15,16] were not able to quantify the discrete behaviour of this layer, rather its properties in relation to the outer, muscular layer. Additionally, none of the referenced *in vivo* studies considered the time- or history-dependent behaviour of the human oesophagus.

The layer-dependent properties of the oesophagus can be established *ex vivo* due to the fact that it is the only visceral organ that can be relatively easily separated into its two main layers after explantation through careful cutting of the loose connective tissue binding the layers together [17,18]. However, as of yet, the passive mechanical properties of the discrete layers of the oesophagus have not been investigated using fresh, human tissue [19–23], apart from by Tøttrup *et al.* [24], who tested the behaviour of only isolated longitudinal and circular smooth muscle from the human oesophagus over three decades ago. There have been many studies investigating the mechanical properties of different animal oesophagi, including rat [25–28], guinea pig [29,30], porcine [31–36], rabbit [37,38] and ovine [39,40], with most conducting a layer-dependent analysis [25,26,28,29,31,33–36,38,39]. Now, animal studies are highly valuable for assessing the direct influence of diseases, e.g. diabetes [41–43], oesophageal varices [44,45] and irritable bowl syndrome [46], with the use of animal models, and for evaluating interventions, e.g. surgical approaches [47,48] and medicinal compounds [43,49,50], as they allow for a very controlled environment and, often, a larger sample size compared with human studies. However, in terms of mechanical behaviour, it has been found that animal soft tissues, including the gastrointestinal tract, can differ significantly from their human counterparts [51–53]. Therefore, as they may not accurately represent the material response of human tissue, experimental findings from animal studies should not ideally be used quantitatively for modelling the human oesophagus for applications within medicine [51]. For instance, layer-dependent models of the human oesophagus provide a means to investigate how a medical device would interact with the different oesophageal layers. However, currently, models developed for devices, such as oesophageal stents, use material parameters determined from animal experimental data [54,55]. Furthermore, tissue-engineered oesophagi are a promising treatment for diseases such as atresia [56,57]. For this, the establishment of the passive mechanical properties of the native human oesophagus can be used to cross-reference the properties of the grown tissue, ensuring the material behaviour is sufficiently close to that of the native [40]. Moreover, quantification of the viscoelastic properties of human organs can be used to increase the realism of surgical simulations, by providing a training technique that is both time-dependent and stress–strain-dependent [58,59]. Therefore, to provide insight and mechanical data for these applications, the layer, direction and time-dependent behaviour of the fresh human oesophagus was explored in this study through a series of increasing stretch-level cyclic tests and stress-relaxation experiments.

For a considerable amount of time, corpses have been used in the training of medical students on anatomy and surgical techniques [60]. Although the benefits of using surgical simulations for the latter purpose are great, surgical simulation systems are currently not developed enough in terms of initial system acquisition cost, force-feedback experience and overall realism to be used widely within the medical population [61,62]. Therefore, cadaveric surgical training is still prominent. An important consideration when using cadavers for this purpose is how they are preserved. There are a number of ways in which this is possible, including fresh-frozen, formalin fixation and Thiel’s method [63], each with their own advantages and disadvantages. Formalin is the traditional solution used for embalming, while Thiel’s method is a more recently developed technique that provides organs with a softer appearance and more realistic colours [64]. It is of value to establish how the cadaver preservation method affects the mechanical behaviour of soft tissues, providing insight into the experience the medical student will have and the differences they might expect between training and surgery on living organs [65]. In regard to current preservation process studies, some have looked into the effect of just Thiel on the mechanical properties of soft tissues [66–68]; however, formalin-preserved cadavers are still used for medical students’ anatomical and surgical training due to their wider availability, lower cost, simpler embalming technique and the little effect it has on the functional anatomical knowledge of students when compared with Thiel [60,65]. Despite this, studies in the literature on the effects of formalin on the material properties of human tissues are few and their findings contradictory [69,70]. Therefore, the current study hopes to provide more insight into the effects of formalin on soft tissues by comparing the embalmed cyclic oesophageal tissue results of the authors’ previous studies [22,23] with the fresh oesophageal tissue results presented here.

To more fully understand the mechanical behaviour of the human oesophagus, the cyclic and stress-relaxation material response of the two main layers of the organ were investigated in this study; the data of which can be used for insight into the function of the different oesophageal layers, for modelling the human oesophagus with respect to its individual layers, and for comparison with the mechanical properties of tissue-engineered oesophagi. To assess the residuals strains of the organ, zero-stress state analysis was also carried out via the radial cut method. Further to this, quantitative analysis of previously acquired histological images of the human oesophagus was conducted to provide insight into the relationship between fibre fraction and the observed material behaviour of the organ’s layers. To this end, fresh and embalmed cyclic results are compared to explore the influence of formalin fixation on the human oesophagus’ mechanical properties.

## 2. Experimental methods

### 2.1. Sample extraction

The three oesophagi used for mechanical characterization within this study were extracted by means of dissection from fresh cadavers at the Laboratoire d’Anatomie Des Alpes Françaises, Grenoble, France. After death and before dissection, the cadavers were stored in a 4°C refrigerated room, and the dissection was performed within 24 h after death.

The precise procedure for dissection can be found in our previous study [22]. However, for Cadaver 3, an extra step was carried out in order to consider the axial prestretch of the human oesophagus. For this, three sections 5 cm apart were marked *in situ* on the organ’s thoracic region using a dissolvable ink pen. After excision, the lengths of these sections were measured. The difference in length of the sections between the oesophagus’ *in situ* and *ex vivo* state was then used to calculate the average (mean) axial prestretch of the thoracic region of the organ.

Once removed from the cadavers, the oesophagi were examined by a medical professional and were deemed healthy. Afterwards, they were taken immediately for sample preparation for the mechanical tests. This study was performed in compliance with French regulations on post-mortem testing, and the protocol was approved by the Ethics, Scientific and Educational Committee of Grenoble Alpes University (CESP de Centre Don du Corps). Due to constraints regarding time and the number of tests carried out, all tests were conducted within 5 days of dissection, during which, other than when being used for testing, the tissue was stored in physiological saline solution (0.9% NaCl) at $4^{\circ }C$.

### 2.2. Histology

Histological analysis was carried out in our previous studies using oesophagi retrieved from cadavers that had been embalmed with formalin solution (ARTHYL) injected into the carotid artery and drained from the jugular vein [22,23]. Samples with a thickness of 3 μm were taken from the transversal and longitudinal planes of the oesophagus with its layers intact. The slides were stained with either Sirius Red, to see all types of muscular and collagen fibres, with Haematoxylin Eosin Saffron (HES), to highlight the nucleic acids and connective tissue (among other collagen), or with Orcein, to show the elastin fibres. Histological images were then taken, an example of which can be seen in Durcan *et al.* [22,23], and were processed in this study to quantify the percentage collagen and elastin fibre content in each plane and layer. These fibres were chosen due to their influence in the mechanical behaviour of soft tissues [71].

Firstly, the images were manually segmented to allow the fibre content of the different layers to be determined. Next, ImageJ [72] was used to process the images and estimate the percentage collagen content from the Sirius Red and HES images, and the percentage elastin content from the Orcein images. For this, the non-tissue background was removed from the images, the contrast was increased, and the area of the whole tissue region was evaluated. Then, the area of colour specific to the fibre of interest was determined. Finally, the percentage fibre content was calculated by dividing the fibre area by the area of the whole tissue region and multiplying the answer by 100. This was repeated for each layer in the longitudinal and transversal histological images.

The final fibre contents were calculated as a mean percentage taken from images from three sections along the thoracic region (top, middle and bottom) across two oesophagi retrieved from cadavers of similar ages to those investigated in this study.

### 2.3. Uniaxial tensile testing

#### 2.3.1. Sample preparation

**Figure 1.**
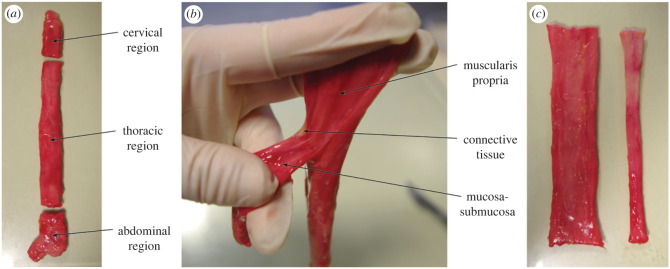
(*a*) Separation of the oesophagus into its three main regions (cervical, thoracic and abdominal). (*b*) Dissection of the two main oesophageal layers. (*c*) Fully separated layers of the human oesophagus showing the unravelled muscularis propria on the left and the tubular mucosa-submucosa on the right.

Upon excision from the human cadavers, the oesophagi were first gently rinsed through the lumen using saline. They were then carefully cleaned of any excess connective tissue and separated into their three main regions (cervical, thoracic and abdominal), as seen in figure 1*a*. At this point, samples were cut for zero-stress state analysis from the oesophagus of Cadaver 3, the protocol of which is described in §2.4. As the region-dependent tensile properties were not being considered in this study, only the thoracic region was used for mechanical testing. This region was chosen for consistency as it comprises the vast majority of the organ and contains a mix of both striated and smooth muscle [73].

Next, the thoracic region of each oesophagus was fully separated into its two main layers. For this, firstly a longitudinal incision was made along the length of the region, cutting through only the muscularis propria layer. This incision allowed access to the connective tissue between the layers, which was then very carefully cut to dissect the two layers. An image taken during the layer separation process can be seen in figure 1*b*, while the fully separated layers can be seen in figure 1*c*. Once separated, the mucosa-submucosa layer was cut along its longitudinal length and unravelled. The layers were then examined visually to determine if any damage, i.e. cuts penetrating partially or fully into the tissue layers, had been incurred during separation, of which there had not. Immediately prior to testing, samples approximately 22.00 × 4.10 mm (length × width) were cut in both the longitudinal and circumferential directions. In between tests, the tissue layers were stored in physiological saline solution at 4°C. The samples were brought to room temperature before testing.

#### 2.3.2. Experimental set-up

**Figure 2.**
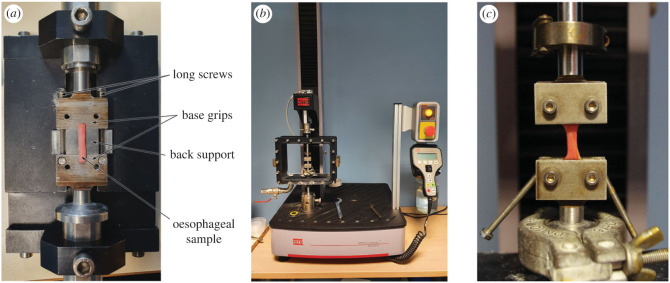
(*a*) Sample positioned on the support ready to be secured within the grips. (*b*) Machine set-up. (*c*) A sample loaded within the machine.

Once cut, the rectangular samples were secured within the grips using the following procedure: first, the samples were laid on the base grips and back support of a special device designed to set up soft tissue samples within the grips, as seen in figure 2*a*. Next, the samples were flattened and aligned as centrally as possible upon the grips. This step often took some time due to the very soft and sticky nature of the tissue. Then, the upper grips were placed on the base grips and the screws tightened using a torque limiter set at 0.5 Nm, to prevent the samples slipping during testing and for consistency across the different samples. After this, the long screws situated either side of the grips, as shown in figure 2*a*, were tightened to create an assembly in which the soft sample could be moved and set up in the mechanical testing machine, an MTS Criterion model C41 (MTS Systems, Minnesota). The assembly was used to position the sample within the machine, as seen in figure 2*b*, by attaching the lower portion of the assembly directly to it and then the upper portion to a highly sensitive 25 N load cell. Once secured within the machine, the long screws situated either side of the grips were loosened to release the assembly. The back support holding the tissue sample in place was then removed. The final set-up of a sample within the machine prior to commencement of the tests can be seen in figure 2*c*. At this point, the width and thickness of the sample were measured at three separate points along its length using callipers, and the average (mean) was used for the calculation of stress (§2.3.5). Tensile deformation was employed via upward traction of the upper grip attached to the crosshead and load cell while the lower grip remained fixed.

#### 2.3.3. Stress-relaxation tests

To investigate the relaxation response and equilibrium stress of each layer of the oesophagus, stress-relaxation tests were conducted, in which the samples were stretched at a rate of $100\%\;s^{-1}$ and held initially for 20 min for the first trial of all layers and directions. The remaining trials were then held for 15 min as the drop in stress during the first trials between the 20 and 15 min mark was only 2%. Due to the limited number of samples available from a single human oesophagus, the stress-relaxation tests were conducted in the form of multi-step tests to extract the greatest amount of information from a single sample. The longitudinal samples for both layers were stretched in increments of 5% deformation and the circumferential samples were stretched in increments of 10% deformation. The tests were stopped once the samples had ruptured. These strain steps were chosen to ensure at least four relaxation steps were completed in each direction before the samples ruptured and, thus, that the equilibrium stress-stretch curve for each direction could be established. They were decided based on the results of the authors’ previous studies on embalmed oesophageal tissue [22,23]. All tests were carried out at ambient temperature and conducted under a uniaxial tensile test condition. The samples were kept moist during the tests via routine spraying with physiological saline solution.

#### 2.3.4. Cyclic tests

Cyclic tests were performed in the form of increasing stretch-level cyclic tests with two cycles per level. This form of test was chosen over a single cycle or a single deformation-level cyclic test to be able to observe the most phenomena while testing the fewest samples. Deformation levels of 10–70% in increments of 10% (stretch levels of 1.1–1.7 with increments of 0.1) were chosen for all samples. The cyclic tests were conducted at two different strain rates, $1\%\;s^{-1}$ and $10\%\;s^{-1}$, to explore any rate-dependent behaviour of the tissue. An average of seven tests per oesophagus, layer, direction and strain rate were conducted. Again, all tests were carried out at ambient temperature and conducted under a uniaxial tensile test condition, and the samples were kept moist during testing via routine spraying with physiological saline solution.

#### 2.3.5. Mechanical characterizations

In this study, the stress is expressed in terms of nominal stress (i.e. first Piola–Kirchhoff stress), which is defined as

2.1 $$P=\frac{F}{A_{0}},$$

where *F* is the applied force and *A*_0_ is the cross-sectional area of the undeformed sample. The experimental strain is expressed in terms of stretch, *λ*, and is defined as *λ* = *l*/*l*_0_, where *l* and *l*_0_ are the current and initial lengths of the sample, respectively. Stretch relates to the nominal strain by ε=λ−1.

### 2.4. Zero-stress state

#### 2.4.1. Sample preparation

**Figure 3.**
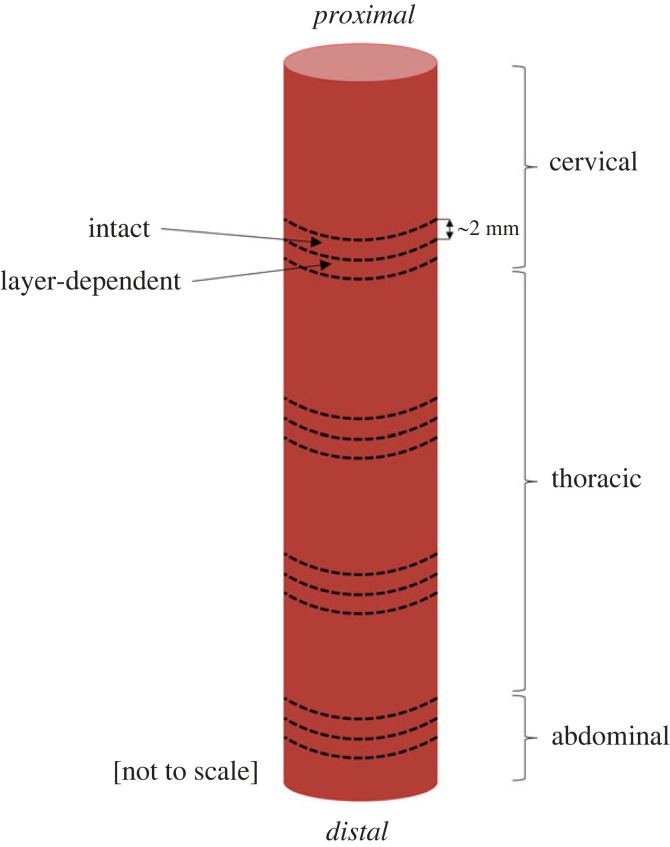
Drawing illustrating the location of the circumferential zero-stress state analysis samples.

The axial prestretch of the oesophagus from Cadaver 3 was measured as described in §2.1. To determine the oesophageal specimen’s residual circumferential strains, ring-like segments were cut from four different locations along the length of the specimen, as seen in figure 3. At each location, two samples approximately 2 mm in length were retrieved. The proximal segment at each location was used to investigate the opening angle and residual strains of the intact wall, while the more distal segment was used for layer-dependent analysis (figure 3). To separate the layers of the rings, careful cutting was administered to the connective tissue binding the layers together, while keeping both the layers in their ring-like forms.

#### 2.4.2. Experimental set-up and protocol

**Figure 4.**
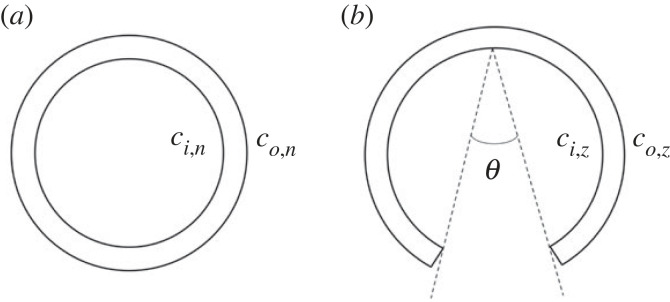
Schematic diagram showing the no-load state (*a*) and the zero-stress state (*b*) of a ring segment, including the definition of the opening angle, *θ*.

A circumferential zero-stress state analysis was conducted as per the protocol outlined in Liu & Fung [74]. The ring-like segments were all submerged in physiological saline solution at room temperature in individual containers. Photographs were taken at this point to be able to determine the dimensions of the samples in the no-load state, as illustrated in figure 4*a*. All images were taken using a Pentax K-5 camera with a 50 mm lens. The segments were then cut radially using surgical scissors while still submerged in the solution. Photographs were taken immediately after the samples were cut, then again at 0.5, 1, 2, 3, 4, 5 and 24 h to assess the time evolution of the opening of the sector. The opening angle, *θ*, as defined in figure 4*b*, was then measured for all the open segment images.

#### 2.4.3. Measurement of circumferential residual strains

The residual circumferential strains were initially measured in terms of Green’s strain using the change in wall dimensions between the no-load and the zero-stress state. The following equations were used to calculate the residual Green’s strain at the inner and outer surfaces, respectively:

2.2 $$e_{i}=\frac{{\left(c_{i,n}/c_{i,z}\right)}^{2}-1}{2}$$

and

2.3 $$e_{o}=\frac{{\left(c_{o,n}/c_{o,z}\right)}^{2}-1}{2},$$

where *c*_*i*,*n*_ and *c*_*i*,*z*_ are the circumference of the inner surface at the no-load and zero-stress state, respectively, and *c*_*o*,*n*_ and *c*_*o*,*z*_ are the circumference of the outer surface at the no-load and zero-stress state, respectively. The definitions of these quantities can be seen schematically in figure 4. The stretch relates to Green’s strain by *e* = (*λ*^2^ − 1)/2.

### 2.5. Experimentation of embalmed tissue

Previous works by the authors consisted of cyclic experimentation of embalmed human cadaveric oesophagi [22,23]. The same protocol as outlined in §2.3.4 was used for the tests, allowing for a direct comparison between the preservation states. In the present study, the fresh tissue cyclic results were compared with the embalmed tissue results for each layer, direction and strain rate.

### 2.6. Statistical analysis

Often, a mean is used to describe the average result of a certain quantity. However, this should only be used if the dispersion of the quantity follows a normal distribution. Therefore, initially, tests were carried out to determine if the quantities of interest in this study were normally distributed. For this, a Shapiro–Wilk test was conducted using IBM SPSS Statistics (v. 27.0) [75] with a significance level, *α*, of *α* = 0.05. The distribution was considered normal if *p* > 0.05. If this was the case, a mean value would be used to represent the quantity. If, however, the quantity was not normally distributed, tests would be carried out to see if its dispersion followed a Fréchet distribution: for this, R statistical software was used [76]. Fréchet distribution was used previously by the authors as (i) it was found that Young’s modulus of all the embalmed tissue results followed this right-skewed distribution [22,23], and (ii) Fréchet distribution is suitable for the application of material properties [77]. The non-normally distributed data was tested for a Fréchet distribution with *α* = 0.05, meaning that if *p* > 0.05, the quantity follows the distribution. In this case, the mode of the Fréchet distribution provides the most representative value, and so if a quantity followed this distribution, the mode would be used for its analysis. To establish differences between groups, an independent non-parametric test was used in the form of the Mann–Whitney *U* test due to the often non-normal distribution of the data.

## 3. Results

### 3.1. Demographics

A total of three fresh oesophagi were tested in this study. The length of the oesophagi from Cadavers 1, 2 and 3 were approximately 24, 28 and 26 cm, respectively. The demographics of the patients and the tests conducted using each oesophagus are outlined in table 1.

**Table 1.** Patient demographics and the tests conducted on each cadaveric specimen.

cadaver	sex	height (cm)	weight (kg)	age (years)	type of tests
1	female	144	42	96	cyclic
2	male	185	78	89	cyclic, stress-relaxation
3	male	170	100	97	cyclic, stress-relaxation, zero-stress state

### 3.2. Histological analysis of the human oesophagus

**Figure 5.**
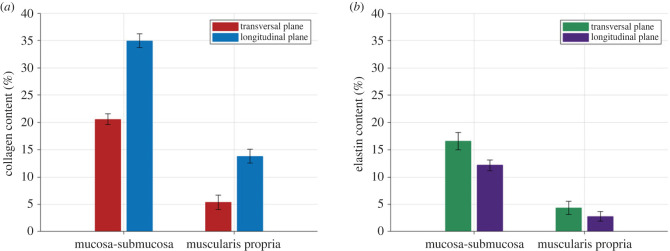
Collagen (*a*) and elastin (*b*) content in the different layers and planes of the human oesophagus determined through histological image analysis.

A qualitative description of the elastin and collagen content of the muscularis propria and mucosa-submucosa can be found in Durcan *et al.* [22] and Durcan *et al.* [23], respectively. Here, the histological images were processed to approximate the amount of collagen and elastin in each layer and direction of the human oesophagus. As both the Sirius Red and HES stains display the collagen of the tissue, the Sirius Red images were analysed first to determine the percentage of collagen in each layer and direction. Then, the transverse sections of the HES images were analysed to validate the percentage of collagen estimated using the Sirius Red images. The validation was successful with only a 5% difference between the two percentages for the mucosa-submucosa, and a 12% difference between the two collagen content values for the muscularis propria. The results of the percentage collagen in each layer and plane determined from the Sirius Red images can be found in figure 5*a*. The results of the Orcein image analysis can be seen in figure 5*b*. As there was only one stain that highlighted the elastin fibres of the tissue, there could be no validation of the elastin content calculation.

### 3.3. Stress-relaxation results

#### 3.3.1. Variation in experimental samples and statistical analysis

The total number of stress-relaxation tests conducted per layer, per direction and per cadaver can be found in table 2. Many factors can contribute towards the dispersion seen among experimental data of soft biological tissues, including heterogeneity of the tissue, different levels of moisture, fluctuations in ambient temperature and variations in sample dimensions. The variations in width and thickness of the stress-relaxation samples are displayed in table 3.

**Table 2.** Number of stress-relaxation tests per layer, per direction, per cadaver.

layer	direction	step in strain (%)	cadaver	tests	total
muscularis propria	longitudinal	5	2	5	*n* = 10
			3	5	
	circumferential	10	2	5	*n* = 9
			3	4	
mucosa-submucosa	longitudinal	5	2	5	*n* = 10
			3	5	
	circumferential	10	2	5	*n* = 10
			3	5

**Table 3.** Mean ± population standard deviation of the sample dimensions for the stress-relaxation experiments.

	muscularis propria		mucosa-submucosa	
	width (mm)	thickness (mm)	width (mm)	thickness (mm)
longitudinal	4.2 ± 0.3	1.8 ± 0.5	4.0 ± 0.2	0.8 ± 0.2
circumferential	4.1 ± 0.3	1.9 ± 0.4	3.9 ± 0.2	0.6 ± 0.3

To establish the time-independent behaviour of the oesophageal layers, the equilibrium stress-stretch points for each test were recorded. There was variability in these points between the different tests for each direction and layer. To obtain the average equilibrium stress-stretch points, outlying tests were removed (maximum two per layer, per direction) and the mean was calculated using the remaining values. These values were then used to plot the equilibrium stress-stretch curve to determine the time-independent response of each layer and direction of the human oesophagus under large deformation, i.e. its hyperelastic behaviour.

#### 3.3.2. Stress-relaxation behaviour

**Figure 6.**
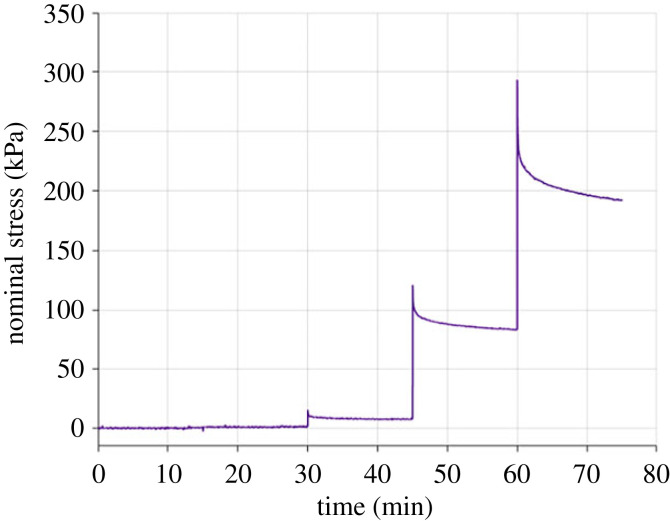
Stress-time example from the multi-step stress-relaxation experiments of the human oesophageal mucosa-submucosa layer in the longitudinal direction.

**Figure 7.**
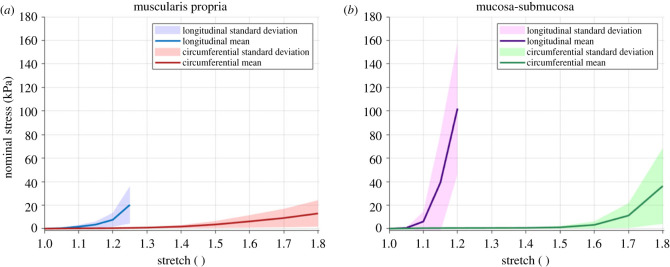
Mean equilibrium stress-stretch curves obtained from the multi-step stress-relaxation experiments of the human oesophageal muscular (*a*) and mucosal (*b*) layers in the longitudinal and circumferential directions, including shaded areas showing the sample standard deviations.

The multi-step stress-relaxation behaviour of fresh human oeosphageal tissue was found to be different depending on the layer and direction. An example of a typical stress-time response can be seen for the longitudinal mucosal layer in figure 6. The mean equilibrium stress-stretch curve for each layer and direction can be found in figure 7, including the standard deviations. In the longitudinal direction, the mucosa-submucosa reveals a substantially greater equilibrium stress for a certain stretch compared with the muscularis propria, particularly from 1.1 stretch onwards. In the circumferential direction, the behaviour of the two layers is similar until 1.4 stretch, at which point the muscularis propria becomes slightly stiffer than the mucosa-submucosa. Then, after 1.6 stretch, the stress of the mucosa-submucosa increases exponentially compared with the muscularis propria. For both layers, the equilibrium stress is greater in the longitudinal direction than in the circumferential direction. The equilibrium stress-stretch results reveal that the time-independent behaviour of the human oesophagus is both layer- and direction-dependent.

### 3.4. Cyclic results

#### 3.4.1. Variation in experimental samples and statistical analysis

**Figure 8.**
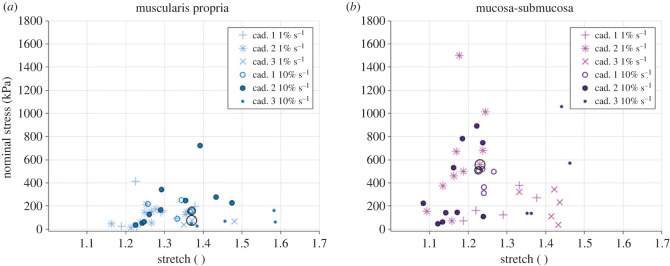
Rupture stress-stretch for each cyclic test conducted in the longitudinal direction of the muscularis propria (*a*) and mucosa-submucosa (*b*) layers, highlighting the dispersion between cadavers and the two strain rates ($1\%\;s^{-1}$ and $10\%\;s^{-1}$). The circled points show the rupture stress-stretch of the curves selected for analysis via statistical means, as outlined in §3.4.1.

The total amount of cyclic tests conducted on the fresh oesophageal layers per direction, strain rate and cadaver can be found in table 4, while table 5 shows the means and standard deviations of the sample thicknesses and widths. Across the three cadavers, there was variation between the tests completed for each layer, direction and strain rate, particularly for the mucosa-submucosa samples. These variations can be visualized in figure 8. It should be noted that, when analysing the results, only one test condition (specific layer, direction and strain rate) showed a significant correlation between days since dissection and maximum stress of the samples, with no significant correlation being found for any of the other seven test conditions (statistical analysis conducted using Spearman rank-order correlation); therefore, it was assumed that time since dissection did not play a substantial role in the variability between samples, for which the heterogeneity inherent to soft tissues was thought to be much more influential.

**Table 4.** Number of cyclic tests per layer, per direction, per strain rate, per cadaver.

layer	direction	strain rate	cadaver	tests	total
muscularis propria	longitudinal	$1\%\;s^{-1}$	1	7	*n* = 20
			2	10	
			3	3	
		$10\%\;s^{-1}$	1	5	*n* = 20
			2	10	
			3	5	
	circumferential	$1\%\;s^{-1}$	1	5	*n* = 20
			2	10	
			3	5	
		$10\%\;s^{-1}$	1	5	*n* = 20
			2	10	
			3	5	
mucosa-submucosa	longitudinal	$1\%\;s^{-1}$	1	5	*n* = 20
			2	10	
			3	5	
		$10\%\;s^{-1}$	1	6	*n* = 20
			2	10	
			3	4	
	circumferential	$1\%\;s^{-1}$	1	5	*n* = 20
			2	10	
			3	5	
		$10\%\;s^{-1}$	1	3	*n* = 20
			2	10	
			3	7

**Table 5.** Mean ± population standard deviation of the sample dimensions for the cyclic experiments across both strain rates ($1\%\;s^{-1}$ and $10\%\;s^{-1}$).

	muscularis propria		mucosa-submucosa	
	width (mm)	thickness (mm)	width (mm)	thickness (mm)
longitudinal	4.1 ± 0.4	2.0 ± 0.4	4.2 ± 0.2	0.9 ± 0.2
circumferential	4.1 ± 0.4	2.4 ± 0.4	4.1 ± 0.3	0.7 ± 0.1

**Figure 9.**
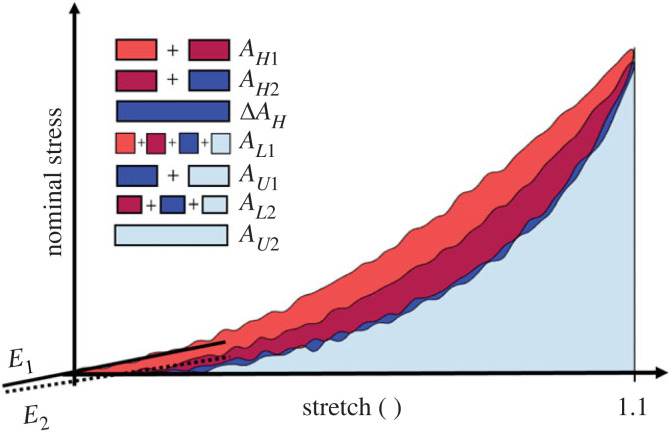
Schematic diagram showing the different characteristics extracted from the two 1.1 cycles, where *E*_1_ = Young′s modulus of the loading curve of the first cycle, *E*_2_ = Young′s modulus of the loading curve of the second cycle, *A*_*H*1_ = hysteresis area of the first cycle, *A*_*H*2_ = hysteresis area of the second cycle, Δ*A*_*H*_ = difference in hysteresis area between the two cycles, *A*_*L*1_ = area under loading curve of the first cycle, *A*_*U*1_ = area under unloading curve of the first cycle, *A*_*L*2_ = area under loading curve of the second cycle and *A*_*U*2_ = area under unloading curve of the second cycle.

**Figure 10.**
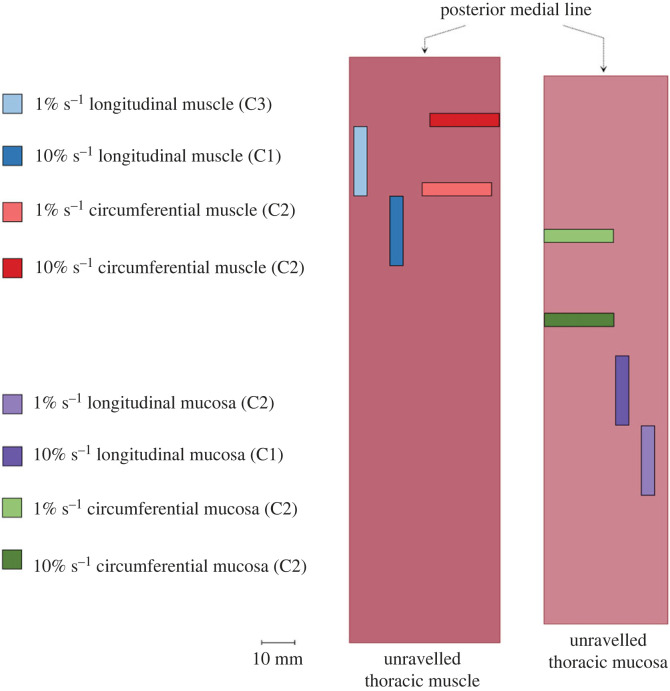
Anatomical destination of the experimental samples retrieved through statistical analysis to represent the cyclic behaviour of the human oesophageal layers, where C1, C2 and C3 denote Cadavers 1, 2 and 3, respectively.

To quantify the variability of the data and to deduce the most representative curve, 11 features (characteristics) were extracted from *λ* = 1.1 of all the cyclic experimental data. This cycle was chosen as it allowed for comparison across all layers, directions and strain rates, as well as between the embalmed and fresh tissue. The characteristics include Young’s modulus of the first loading curve, Young’s modulus of the second loading curve, the hysteresis of the first cycle, the hysteresis of the second cycle, the difference between the two hystereses, the rupture stretch, *λ*_rup_, the maximum stress, *P*_max_, and the areas under the loading and unloading curves for both the first and second cycle of *λ* = 1.1; the definitions of which can be seen in figure 9. The distributions of all these features were established by first testing if they were normally distributed using a Shapiro–Wilk test in SPSS. If the characteristic was normally distributed for a certain layer, direction and strain rate, the mean of the value would be taken as the most representative. Next, if the distribution was not normal, the characteristic would be tested to see if it followed a Fréchet distribution. It was found that all non-normally distributed characteristics followed a Fréchet distribution (*p*-values of which will be presented for the distribution of *E*_1_ in §4): therefore, the mode of the Fréchet distribution for these characteristics was taken as the most representative value. Then, the mean or mode of each characteristic was compared with the characteristic values of each individual test. The test with the highest number of characteristics close to the mean or mode was chosen to represent the behaviour of the specific layer, direction and strain rate. These experimental curves were used for analysis of the cyclic behaviour of the fresh human oesophageal layers and will be presented in the subsequent graphs; the anatomical destinations of which can be seen in figure 10. Additionally, the differences between groups were measured regarding all the aforementioned characteristics of the *λ* = 1.1 cycles using a Mann–Whitney *U* test due to not all characteristics being normally distributed.

#### 3.4.2. Presentation of cyclic results

As described in §2.3.4, the cyclic tests were carried out with two cycles per stretch level, and both cycles have been presented here. Moreover, the longitudinal samples ruptured before *λ* = 1.7, therefore only the full cycles have been presented for this direction, i.e. the complete cycles undergone before the samples ruptured.

#### 3.4.3. Cyclic stress–strain behaviour

**Figure 11.**
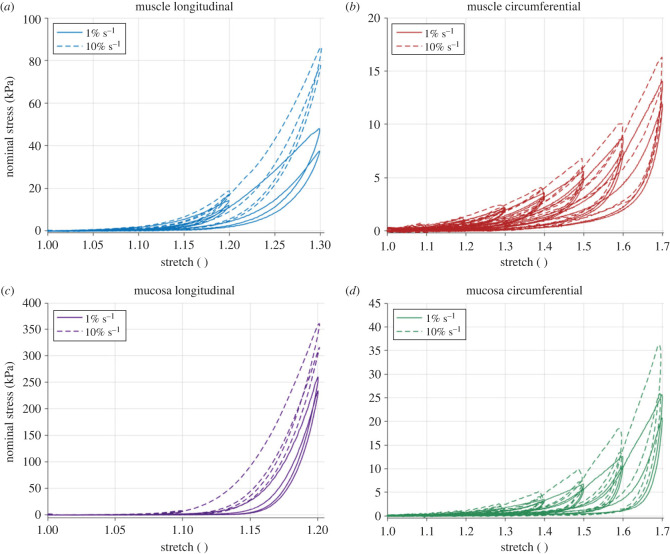
Cyclic oesophageal tissue results showing the difference between the $1\%\;s^{-1}$ and $10\%\;s^{-1}$ response for the muscularis propria in the longitudinal (*a*) and circumferential (*b*) directions, and the mucosa-submucosa in the longitudinal (*c*) and circumferential (*d*) directions.

**Figure 12.**
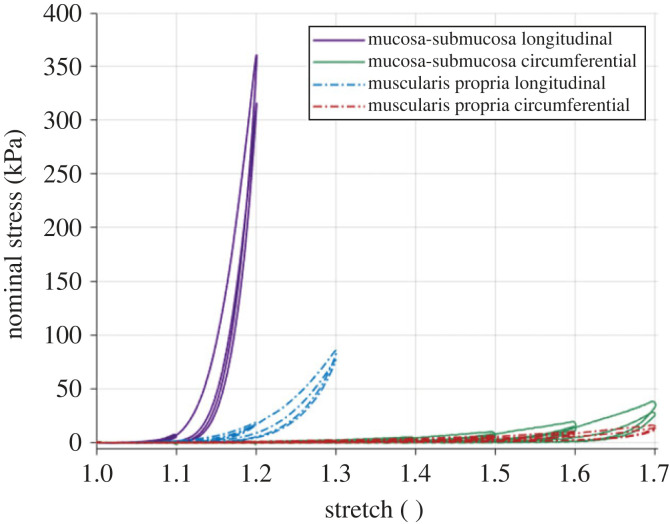
Comparison between the $10\%\;s^{-1}$ cyclic experimental results of the muscularis propria and the mucosa-submucosa of the human oesophagus in the longitudinal and circumferential directions.

Figure 11 shows the rate-dependent cyclic results of the muscularis propria and mucosa-submucosa in the longitudinal and circumferential directions. Both layers present highly anisotropic behaviour, with greater stiffness and earlier rupture in the longitudinal directions compared with the circumferential directions. Strain rate-dependent behaviour is also apparent with an increase in strain rate resulting in an increase in stiffness for both layers and directions. Figure 12 presents a layer comparison for the $10\%\;s^{-1}$ results. In the longitudinal direction, past 1.07 stretch, the stress of the mucosa-submucosa is much higher than that of the muscular layer. The mucosal layer also ruptures earlier than the muscularis propria in this direction. While the difference in stiffness between the two layers is not as great for the circumferential direction compared with the longitudinal direction, it can be seen that after 1.4 stretch the stiffness of the mucosa-submucosa layer is greater than the muscularis propria. Permanent deformations of the cyclic results were also found to be layer-dependent in that there was greater permanent set in the mucosa-submucosa than the muscularis propria when comparing across each direction. To determine this, the inelastic strains present after each cycle were plotted against the previous maximum stretch the sample was subjected to (see Durcan *et al.* [23] for more detail); the graphs of which will be presented later in §4. The results of the statistically significant layer differences, strain rate differences and direction differences in terms of the 11 characteristics (§3.4.1) can be seen in tables 7–9 in the appendix, respectively.

### 3.5. Opening angle and residual strains

**Figure 13.**
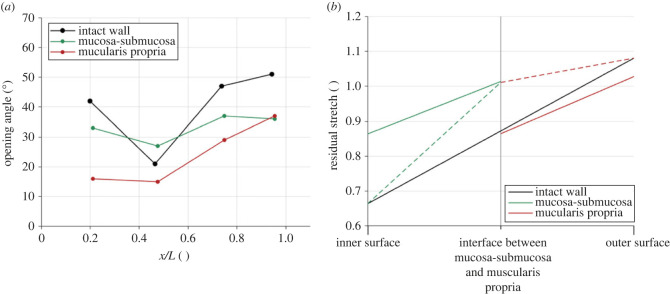
(*a*) Opening angle as a function of axial length along the oesophagus, where *x*/*L* = 0 is the proximal end and *x*/*L* = 1 is the distal end. (*b*) Circumferential residual stretch of the thoracic region of the human oesophagus throughout the tissue wall, where the solid lines show the residual stretches of the intact wall or separated layers, and the dashed green and red lines represent the residual strains of the mucosa-submucosa and muscularis propria, respectively, calculated by considering their inner and outer surfaces within the intact wall samples.

The average axial stretch of the oesophagus from Cadaver 3 from its *ex vivo* state to its *in situ* position was found to be 1.06, meaning that the human oesophagus *in vivo* is under a slight axial tension compared with its zero-stress state. For the circumferential zero-stress state analysis, the time-dependent investigation showed that most of the creep of the samples occurred between 0 and 0.5 h, however, that some slow creep occurred until 24 h. Therefore, the opening angle and residual strain measurements presented here are from the photographs taken at 24 h. The opening angle varies along the length of the oesophagus, and between the different layers, as can be seen in figure 13*a*. The results reveal that there is a greater opening angle in the mucosa-submucosa layer than the muscularis propria along the majority of the oesophagus apart from in the abdominal region. The circumferential residual strains, as displayed in figure 13*b*, show that while the layers are intact, the mucosa-submucosa of the thoracic region is mainly under compression (dashed green line), whereas the muscularis propria is fully in tension (dashed red line). The values in figure 13*b* are the average of both thoracic locations investigated. The residual strains of the separated layers (solid coloured lines) show the typical inner surface under compression, outer surface in tension relationship. The magnitude of these residual strains do not differ much between the separated layers, which is similar to the finding of Zhao *et al.* [78] for the pig oesophagus.

## 4. Comparison of fresh and formalin-embalmed tissue

In the authors’ previous studies, cyclic experimentation was carried out on the two main layers of the oesophagus using organs retrieved from formalin-embalmed cadavers [22,23]. The same experimental protocol was used as in this study, allowing for a direct comparison between the fresh and embalmed preservation states.

The same characteristics as outlined in §3.4.1 were compared between the embalmed and fresh cyclic results for each layer, direction and strain rate using the Mann–Whitney *U* test. It was found that for almost all of the characteristics considered, the magnitude was statistically higher for the embalmed tissue compared with the fresh tissue, the results of which can be found in table 10 in the appendix. The rupture stretch, however, was statistically higher for the fresh tissue compared with the embalmed tissue. Table 6 presents the average Young’s modulus for the $1\%\;s^{-1}$ cyclic results for both fresh and embalmed tissue, revealing a greater increase in stiffness caused by embalming for the mucosa-submucosa compared with the muscularis propria.

**Table 6.** Mode and range of Young’s modulus for each layer and direction from the first cycle (*E*_1_) of the $1\%\;s^{-1}$ cyclic fresh and formalin-embalmed results, and *p*-values for testing if the distribution of Young’s moduli followed a Fréchet distribution (embalmed results as previously reported in Durcan *et al.* [22,23]). Young’s modulus of the fresh circumferential muscularis propria did not follow a Fréchet distribution and was normally distributed, therefore, for this layer, direction and preservation state, the mean, standard deviation and *p*-value for the Shapiro–Wilk test are presented.

layer	direction	pres. state	mode (kPa)	range (kPa)	*p*-value
muscularis propria	longitudinal	fresh	7.94	2.67–20.7	0.722
		embalmed	32.2	6.0–266	0.367
	circumferential	fresh	*mean:*	*standard deviation:*	*normally distributed:*
			3.45	±0.99	0.141
		embalmed	16.1	3.7–81.5	0.808
mucosa-submucosa	longitudinal	fresh	8.36	2.57–24.8	0.926
		embalmed	93.5	25.8–331	0.247
	circumferential	fresh	1.89	0.83–3.52	0.858
		embalmed	38.4	21.2–47.5	0.783

**Figure 14.**
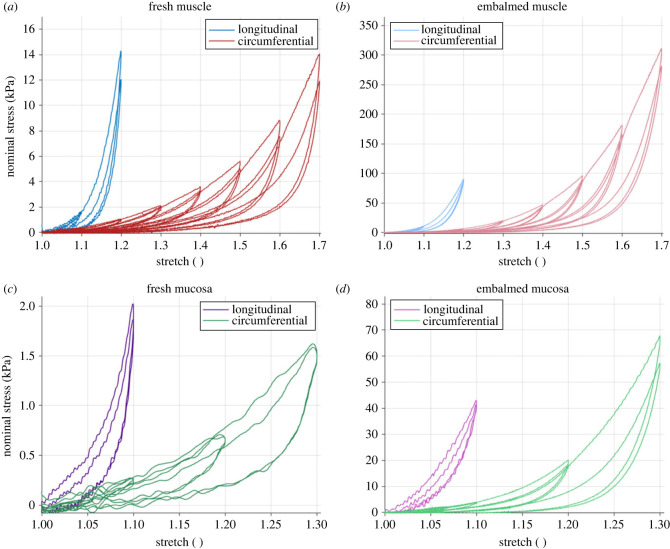
Cyclic experimental results at $1\%\;s^{-1}$ for the fresh (*a*) and formalin-embalmed (*b*) oesophageal muscularis propria, and fresh (*c*) and formalin-embalmed (*d*) oesophageal mucosa-submucosa.

Figure 14 shows that when the axes are scaled accordingly, the anisotropic properties across the preservation states are similar. The anisotropy of stiffness and nonlinearity of each direction, as well as the degree of hysteresis and stress-softening, remain relatively consistent despite the change in magnitude of these phenomena between the embalmed and fresh tissue, suggesting that, apart from rupture stretch, embalming proportionally increases the mechanical properties of oesophageal tissue. It should be noted that due to the limit of the sensor and the softness of the preservation state, there was more noise at lower stretches with the fresh tissue compared with the embalmed tissue, particularly for the mucosa-submucosa layer in the circumferential direction (figure 14*c*).

**Figure 15.**
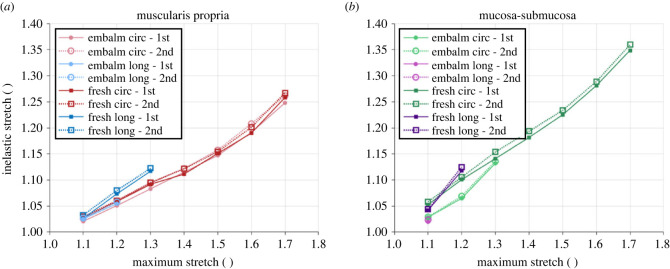
Permanent set from the $1\%\;s^{-1}$ cyclic results of the fresh and formalin-embalmed oesophageal muscularis propria (*a*) and mucosa-submucosa (*b*), including the differences between the first and second cycles.

The differences in permanent set (defined in §3.4.3) were also analysed between the fresh and embalmed tissue cyclic results. Figure 15 shows a comparison between the permanent deformations of the two preservation states. It can be seen that the degree of damage of the fresh circumferential muscular layer was almost identical to that of the embalmed circumferential muscular layer, while the fresh longitudinal muscle, longitudinal mucosa and circumferential mucosa all had greater permanent set than their embalmed equivalents.

## 5. Discussion

The experimental findings of this study provide new insight into the passive mechanical behaviour of the fresh human oesophagus, particularly regarding its layer- and time-dependent properties. The results established the human oesophagus as an anisotropic, visco-hyperelastic material with discrete properties within each layer. Moreover, zero-stress state analysis revealed residual strains in the no-load state of the tissue. Furthermore, determination of the collagen and elastin content of each layer and direction from previously acquired histological images [22,23] showed a greater collagen and elastin content in the mucosa-submucosa than the muscularis propria across both directions, which is in line with similar studies on animal tissue [37].

The equilibrium stress-stretch behaviour and the cyclic results revealed the longitudinal direction to be stiffer than the circumferential direction for both layers of the oesophagus. The circumferential direction also ruptured at a much higher strain than the longitudinal direction for both layers. These behaviours may be related to the oesophagus’ physiological function where more compliance is required in the circumferential direction due to distensions caused by fluid boli of varying size, while greater stiffness in the longitudinal direction supports the function of the longitudinal muscle fibres in carrying out longitudinal shortening during peristalsis, which reduces the work needed by the circular muscle fibres to move the bolus [79]. Microstructurally, there was greater collagen in the longitudinal direction compared with the circumferential direction, and greater elastin in the circumferential direction than in the longitudinal direction for both the mucosal and muscular layers. This histological distribution may explain the anisotropic behaviour of the tissue, as collagen is associated with withstanding tensile loads [80] and elastin allows soft tissues to undergo repetitive loading [81]. The layer-dependent differences also correlate with the histological findings and the role that collagen plays in the mechanical behaviour of soft tissues [71]. Furthermore, the nonlinear response of the tissue layers is associated with the function of the oesophagus in that compliance it required at low stretches to allow for the passage of the bolus, while stiffening occurs at higher stretches to impede over-dilatation. It is thought that the muscularis propria supports the majority of the load at low intraluminal pressures, while the mucosa-submucosa stiffens quickly when the diameter of the oesophagus is approximately double its undeformed size [7,25]. This is inline with the cyclic results presented here in that, in the circumferential direction, the mucosa-submucosa strain hardens at a higher stretch than the muscular layer, as well as, at high stretches, is stiffer than the muscular layer. *In vivo*, the mucosa-submucosa layer is folded and so would only undergo the circumferential loads experienced in the tension experiments carried out here once the layer was distended so far that it unfolded. At this point, the higher stiffness of the mucosa-submucosa would take the load and prevent over-dilatation of the organ.

The main *ex vivo* studies on the human oesophagus conducted uniaxial tensile tests on the intact tissue wall [19,20]. Vanags *et al.* [20], who studied the direction-dependent behaviour of the organ, found the mechanical properties of the human oesophagus to be anisotropic, following the same relationship as the findings of this study: the stress and Young’s modulus were greater in the longitudinal direction, while the strain the tissue could be subjected to before rupture was higher in the circumferential direction. They also investigated the effect of age on the mechanical properties of the human oesophagus [20]. From their findings, it could be expected that Young’s moduli for the oesophagus determined in this study might be greater than for younger oesophageal tissue due to the high ages of the cadavers tested (table 1). Furthermore, Vanags and coworkers [20] conducted distension tests of oesophagi with their layers intact to explore the organ’s mechanism of rupture; however, they did not present any stress–strain plots of their results [20].

Sommer *et al.* [39] studied the mechanical behaviour of ovine oesophagi and carried out tests with a variety of loading modes. They found the inflation-extension behaviour of ovine oesophagi with their layers intact to be direction-dependent, with the longitudinal direction being stiffer than the circumferential direction. This finding agrees with the other inflation-extension tests conducted on intact oesophagi from rats [42] and rabbits [38], as well as the results of this study. Comparing the layer-dependent uniaxial tension results of ovine oesophagi from Sommer *et al.* [39] with those presented here, there was similar maximum stress in the longitudinal direction across both layers, and a similar strain hardening for the muscular layer. However, for the mucosal layer, strain hardening occurred at 1.4 stretch for the ovine oesophagi compared with around 1.1 stretch for the human oesophagi in this study. The circumferential direction of both layers proved to be a lot stiffer for ovine tissue compared with human tissue despite conducting their tests at a slower strain rate: the maximum stress found for ovine oesophagus in this direction was in the range of 200–350 kPa, while in the current study was around 10–40 kPa. Similar layer-dependent, uniaxial tensile tests were carried out by Yang *et al.* [31] on porcine oesophagi, for which the stress in both directions was substantially higher than in this study. While they found the longitudinal direction to be stiffer than the circumferential direction, strain hardening across both layers occurred later in the longitudinal direction and earlier in circumferential direction compared with the current study, suggesting porcine oesophageal tissue to be less direction-dependent than human tissue. These differences could be due to differences in the species’ digestive systems [51], as well as slight variations in the experimental technique and set-up [82].

Layer-dependent distension tests of animal oesophagi performed *ex vivo* were found to agree with the results of this study in that, particularly at higher strains, the mucosa-submucosa was stiffer than the muscularis propria [26,28,36,37,42,83]. This, however, differs from the findings of Frøkjær *et al.* [16] who performed *in vivo* distension tests on healthy humans. They found the stiffness of the oesophagus to be lowest at the inner, mucosal surface across all strains. This discrepancy is thought to be due to the fact that mucosal layer is folded *in vivo* and so most of the stiffness during the tests would be provided by the muscular layer [16]. Therefore, the results of the current study can be used to develop material models of the oesophagus which take into account the discrete behaviour of the layers, while the findings of Frøkjær and coworkers [16] would be ideal in contributing towards the validation of finite-element models of the human organ [84]. While it may be expected that, due to the high ages of cadavers tested in this study [20] and the *ex vivo* test condition [85], the stiffness of the fresh oesophageal tissue tested here would be a lot greater than that found by Frøkjær *et al.* [16], this was not the case, with Young’s moduli (from volunteers with an average age of 37 years) being well within the same order of magnitude: 1.9–3.5 versus 2.5–5 kPa, respectively. Despite the similarities in Young’s moduli between *in vivo* oesophageal findings and the uniaxial tensile results presented here, the distension behaviour of the human oesophagus is of great interest due to it being a tubular organ undergoing distensions *in vivo*. Therefore, future experiments of inflation-extension tests on the separated layers of the human oesophagus, as well as the intact wall, should be performed in order to study, quantify and model its material behaviour in more a physiological-like way. Furthermore, an increase in individual oesophagi tested and the testing of younger tissue would provide a more thorough understanding of the mechanical response of the human oesophagus across the wider population. Additionally, due to the low number of samples per test condition from a statistical point of view, the sample size might not be large enough to properly establish the differences in characteristics between the various strain rates, layers, directions and preservation states. Therefore, a greater sample size in terms of oesophageal specimens as well as individual samples would increase the overall robustness of the statistical analysis and the conclusions drawn. It should be noted that another limitation of this study is that the muscular layer samples were taken from any point within the thoracic region, and were not differentiated based on composition of smooth or striated muscle tissue, which is known to vary along the length of the region [73]. This assumption was taken to reduce the number of variables investigated. However, as there are fundamental differences in microstructural arrangement between smooth and striated muscle, smooth muscle may be more elastic than striated muscle [86], and, in turn, properties such as rupture stretch and permanent set could increase and decrease, respectively, as one moves from the proximal to the distal end of the oesophageal muscular layer. Therefore, in future experimental studies on the human oesophagus, the impact of the varying ratios of striated and smooth muscle throughout the organ on its mechanical behaviour should be considered and investigated.

It was once assumed that the no-load state of human tissues was also its stress-free state. However, it was determined that since unloaded, ring-like cross-sectional segments of the arterial wall sprung open upon a radial cut, there must exist residual stresses and strains within arteries [87–90]. Since then, residual strains and stresses have been found in different tissues of the human body, including aortic valve leaflets [91] and the ureter [92]. Outside of this work, however, only the residual strains present in rabbit [37,38,93], rat [26,27,83], porcine [36,78] and guinea pig [29,30,94] oesophagi have been determined. Gregersen *et al.* [94] found that the inclusion of residual strains within the guinea pig oesophagus provided more accurate strain measures at its luminal surface, while Holzapfel *et al.* [95] found that residual stresses in arteries gave way to a more homogenized stress distribution throughout the arterial wall. The zero-stress state analysis in this study showed there to be circumferential residual strains and axial prestretch in the oesophagus’ no-load state [93], therefore, these should be considered when conducting finite-element modelling of the human oesophagus [96]. In this study, however, only the residual strains of the oesophagus from Cadaver 3 were considered. Therefore, for a more robust quantification of the human oesophageal residual strains, more specimens should be taken into account.

The results presented here regarding the effects of embalming on the human oesophagus may be used by medical students to adjust their expectations when performing practice surgery on cadavers fixed in formalin. It can be seen from the results that the type of nonlinear behaviour is comparable, particularly in regard to the oesophageal layers’ anisotropic relationship and point of strain-hardening, however, that the degree of stiffness across the two preservation states is different. When cutting into embalmed tissue, for instance, the initial stiffness would be approximately four times higher for the muscular layer and 16 times higher for the mucosa-submucosa layer compared with fresh tissue. Additionally, embalmed tissue may rupture at a lower strain, particularly the muscular layer.

As mentioned in §1, the findings in the literature on the effects of formalin preservation on the mechanical properties of soft tissues are contradictory. The understanding of Hohmann *et al.* [69] is that the cross-links formed between the collagen molecules caused by formalin solution increases the stiffness of tissues, while Girard *et al.* [70] believes there is a balance between partial denaturing of collagen and the formation of collagen cross-links, with partial denaturing of the fibres explaining why they found a decrease in stiffness for formalin-preserved tissue when compared with fresh tissue. In the current study, the increase in stiffness caused by embalming agrees with the conclusion of Hohmann *et al.* [69], implying that the cross-links formed by formalin may be more predominant in affecting the mechanical properties of human soft tissues than the partial denaturing of collagen [70]. The fact that the stiffness increase between the fresh and embalmed tissue is greater for the more collagen-rich layer, the mucosa-submucosa, further supports the theory that the formation of collagen cross-links causes the difference in stiffness observed between the two preservation states.

When comparing the same stretch levels for each layer and preservation state at $1\%\;s^{-1}$ (figure 14), it can be seen that the cyclic behaviour of the oesophageal layers in terms of stiffness, hysteresis and stress-softening in the longitudinal and circumferential directions is very similar between the two states, with the main difference being the vast change in magnitude of these mechanical properties (see table 10 in the appendix). Now, the investigation into the influence of collagen cross-links is becoming increasingly popular within the literature [97–101]; this includes the recent development of materials models that take into account collagen cross-link density and orientation [102,103]. It is known that an increase in cross-links leads to an increase in tensile strength [104–106]; however, does the relationship between embalmed and fresh tissue seen in this study suggest that the density of collagen cross-links has a much greater influence on the mechanical behaviour of soft tissues than was once thought, including its viscoelastic and stress-softening properties? If the behaviour between the two directions is the same, with just the magnitude of properties increasing with increasing cross-links caused by embalming, could the properties that are seen, even in the fresh tissue, be heavily influenced by the orientation and density of cross-links? Furthermore, the degree by which the permanent set decreased from the fresh tissue to the embalmed tissue was correlated with the amount of collagen in each layer and direction. The more collagen in a specific layer and direction, the greater the decrease in permanent deformations of the embalmed tissue compared with the fresh tissue. Could this mean that an increase in collagen cross-links causes a resistance to permanent set? Mass spectrometry should be conducted to contrast the collagen cross-link density between fresh and embalmed tissue to determine more concretely if they are the main reason for the differences in properties seen [99].

## 6. Conclusion

The findings of this study present a novel understanding of the layer-dependent mechanical behaviour of the human oesophagus. The results presented here are key in comprehending the relationship between the organ’s material properties and physiological function. They also provide experimental data that can be used in a multitude of biomedical engineering applications, for example, to model the behaviour of the tissue layers to help improve the design of oesophageal stents. Overall, the stress–strain response of the oesophagus was found to be highly anisotropic, with greater stiffness and earlier rupture in the longitudinal direction compared with the circumferential direction for both layers. Regarding the oesophagus’ layer-dependent behaviour, at higher strains and across both directions, the mucosa-submucosa was stiffer than the muscularis propria. Additionally, the study explored how embalming impacts the material behaviour of the oesophagus. Formalin fixation was found to increase the degree of stiffness, hysteresis and stress-softening significantly when compared with fresh tissue. This was proposed to be due to an increase in collagen cross-links brought about by the formalin solution. Further research into the effects of embalming, along with a quantification of their density, may shed more light on the influence of collagen cross-links on the overall mechanical behaviour of human soft tissues. Future work by the authors includes constitutively modelling the experimental data presented here to provide a set of material parameters that may be used directly by the readers.

## Data Availability

This article has no additional data.

## Appendix A

**Table 7.** Statistical differences in characteristics between the layers (muscularis propria and mucosa-submucosa) for both the fresh and formalin-embalmed tissue. Blank cells indicate no statistical significance between the two groups, ‘—’ mean that a statistical test is not relevant, and filled cells indicate the layer for which the characteristic is statistically higher than for the other layer, including the respective *p*-value.

		characteristic										
pres. state	test condition	*E*_1_	*E*_2_	*A*_*H*1_	*A*_*H*2_	Δ*A*_*H*_	*λ*_rup_	*P*_max_	*A*_*L*1_	*A*_*U*1_	*A*_*L*2_	*A*_*U*2_
fresh	$1\%\;s^{-1}$ long						muscle	mucosa				
							*p* = 0.020	*p* = 0.001				
	$10\%\;s^{-1}$ long	mucosa		mucosa	mucosa	mucosa	muscle		mucosa	mucosa	mucosa	
		*p* = 0.037		*p* = 0.001	*p* < 0.000	*p* = 0.001	*p* < 0.000		*p* = 0.003	*p* = 0.030	*p* = 0.004	
	$1\%\;s^{-1}$ circ			mucosa	mucosa	mucosa	—		mucosa			
				*p* < 0.000	*p* < 0.000	*p* < 0.000			*p* = 0.045			
	$10\%\;s^{-1}$ circ	mucosa	mucosa	mucosa	mucosa	mucosa	—		mucosa	mucosa	mucosa	mucosa
		*p* < 0.000	*p* < 0.000	*p* < 0.000	*p* < 0.000	*p* = 0.001			*p* < 0.000	*p* < 0.000	*p* < 0.000	*p* < 0.000
embalmed	$1\%\;s^{-1}$ long		mucosa				muscle	mucosa		mucosa	mucosa	mucosa
			*p* = 0.001				*p* = 0.041	*p* = 0.034		*p* = 0.014	*p* = 0.031	*p* = 0.015
	$10\%\;s^{-1}$ long											
	$1\%\;s^{-1}$ circ			muscle	muscle	muscle						
				*p* = 0.039	*p* = 0.029	*p* = 0.041						
	$10\%\;s^{-1}$ circ			muscle		muscle	mucosa					
				*p* = 0.016		*p* = 0.006	*p* = 0.039

**Table 8.** Statistical differences in characteristics between the strain rates ($1\%\;s^{-1}$ and 10% s^−1^) for both the fresh and formalin-embalmed tissue. Blank cells indicate no statistical significance between the two groups, ‘—’ mean that a statistical test is not relevant, and filled cells indicate the strain rate for which the characteristic is statistically higher than for the other strain rate, including the respective *p*-value.

pres. state	test cond.	characteristic										
		*E*_1_	*E*_2_	*A*_*H*1_	*A*_*H*2_	Δ*A*_*H*_	*λ*_rup_	*P*_max_	*A*_*L*1_	*A*_*U*1_	*A*_*L*2_	*A*_*U*2_
fresh	long muscle				$10\%\;s^{-1}$		$10\%\;s^{-1}$	$10\%\;s^{-1}$				
					*p* = 0.018		*p* = 0.011	*p* = 0.037				
	circ muscle	$1\%\;s^{-1}$	$1\%\;s^{-1}$	$10\%\;s^{-1}$	$10\%\;s^{-1}$	$10\%\;s^{-1}$	—		$10\%\;s^{-1}$	$1\%\;s^{-1}$	$10\%\;s^{-1}$	
		*p* < 0.000	*p* < 0.000	*p* < 0.000	*p* < 0.000	*p* < 0.000			*p* < 0.000	*p* = 0.029	*p* < 0.000	
	long mucosa											
	circ mucosa		$1\%\;s^{-1}$	$10\%\;s^{-1}$	$10\%\;s^{-1}$	$10\%\;s^{-1}$	—		$10\%\;s^{-1}$	$10\%\;s^{-1}$	$10\%\;s^{-1}$	$10\%\;s^{-1}$
			*p* = 0.041	*p* < 0.000	*p* < 0.000	*p* = 0.001			*p* < 0.000	*p* < 0.000	*p* < 0.000	*p* < 0.000
embalmed	long muscle		$10\%\;s^{-1}$									
			*p* = 0.025									
	circ muscle											
	long mucosa											
	circ mucosa		$1\%\;s^{-1}$	$10\%\;s^{-1}$	$10\%\;s^{-1}$							
			*p* = 0.021	*p* = 0.009	*p* = 0.001

**Table 9.** Statistical differences in characteristics between the directions (longitudinal and circumferential) for both the fresh and formalin-embalmed tissue. Blank cells indicate no statistical significance between the two groups , ‘—’ mean that a statistical test is not relevant, and filled cells indicate the direction for which the characteristic is statistically higher than for the other direction, including the respective *p*-value.

Pres. state	Test cond.	characteristic										
		*E*_1_	*E*_2_	*A*_*H*1_	*A*_*H*2_	Δ*A*_*H*_	*λ*_rup_	*P*_max_	*A*_*L*1_	*A*_*U*1_	*A*_*L*2_	*A*_*U*2_
Fresh	muscle $1\%\;s^{-1}$	long	long	long	long	long	—	—	long	long	long	long
		*p* < 0.000	*p* < 0.000	*p* < 0.000	*p* < 0.000	*p* < 0.000			*p* < 0.000	*p* < 0.000	*p* < 0.000	*p* < 0.000
	muscle $10\%\;s^{-1}$	long	long	long	long	long	—	—	long	long	long	long
		*p* < 0.000	*p* < 0.000	*p* < 0.000	*p* < 0.000	*p* < 0.000			*p* < 0.000	*p* < 0.000	*p* < 0.000	*p* < 0.000
	mucosa $1\%\;s^{-1}$	long	long	long	long	long	—	—	long	long	long	long
		*p* < 0.000	*p* < 0.000	*p* < 0.000	*p* < 0.000	*p* < 0.000			*p* < 0.000	*p* < 0.000	*p* < 0.000	*p* < 0.000
	mucosa $10\%\;s^{-1}$	long	long	long	long	long	—	—	long	long	long	
		*p* < 0.000	*p* = 0.001	*p* < 0.000	*p* = 0.001	*p* < 0.000			*p* < 0.000	*p* = 0.012	*p* < 0.000	
Embalmed	muscle $1\%\;s^{-1}$	long	long	long	long	long	circ	long	long	long	long	long
		*p* = 0.001	*p* = 0.009	*p* < 0.000	*p* < 0.000	*p* < 0.000	*p* = 0.003	*p* < 0.000	*p* < 0.000	*p* < 0.000	*p* < 0.000	*p* < 0.000
	muscle $10\%\;s^{-1}$	long	long	long	long	long	circ	long	long	long	long	long
		*p* < 0.000	*p* < 0.000	*p* < 0.000	*p* < 0.000	*p* < 0.000	*p* = 0.001	*p* < 0.000	*p* < 0.000	*p* < 0.000	*p* < 0.000	*p* < 0.000
	mucosa $1\%\;s^{-1}$	long	long	long	long	long	circ	long	long	long	long	long
		*p* < 0.000	*p* < 0.000	*p* < 0.000	*p* < 0.000	*p* < 0.000	*p* < 0.000	*p* < 0.000	*p* < 0.000	*p* < 0.000	*p* < 0.000	*p* < 0.000
	mucosa $10\%\;s^{-1}$	long	long	long	long	long	circ	long	long	long	long	long
		*p* < 0.000	*p* < 0.000	*p* < 0.000	*p* < 0.000	*p* < 0.000	*p* < 0.000	*p* < 0.000	*p* < 0.000	*p* < 0.000	*p* < 0.000	*p* < 0.000

**Table 10.** Statistical differences in characteristics between the preservation states (fresh and formalin-embalmed) for both the muscularis propria and the mucosa-submucosa. Blank cells indicate no statistical significance between the two groups, and filled cells indicate the preservation state for which the characteristic is statistically higher than for the other preservation state, including the respective *p*-value.

		characteristic										
layer	test cond.	*E*_1_	*E*_2_	*A*_*H*1_	*A*_*H*2_	Δ*A*_*H*_	*λ*_rup_	*P*_max_	*A*_*L*1_	*A*_*U*1_	*A*_*L*2_	*A*_*U*2_
muscle		embalm	embalm	embalm	embalm	embalm		embalm	embalm	embalm	embalm	embalm
	$1\%\;s^{-1}$ long	*p* < 0.000	*p* < 0.000	*p* < 0.000	*p* < 0.000	*p* < 0.000		*p* < 0.000	*p* < 0.000	*p* < 0.000	*p* < 0.000	*p* < 0.000
		embalm	embalm	embalm	embalm	embalm	fresh	embalm	embalm	embalm	embalm	embalm
	$10\%\;s^{-1}$ long	*p* < 0.000	*p* < 0.000	*p* < 0.000	*p* < 0.000	*p* < 0.000	*p* = 0.021	*p* = 0.006	*p* < 0.000	*p* < 0.000	*p* < 0.000	*p* < 0.000
		embalm	embalm	embalm	embalm	embalm	fresh	embalm	embalm	embalm	embalm	embalm
	$1\%\;s^{-1}$ circ	*p* < 0.000	*p* < 0.000	*p* < 0.000	*p* < 0.000	*p* < 0.000	*p* < 0.000	*p* < 0.000	*p* < 0.000	*p* < 0.000	*p* < 0.000	*p* < 0.000
		embalm	embalm	embalm	embalm	embalm	fresh	embalm	embalm	embalm	embalm	embalm
mucosa	$10\%\;s^{-1}$ circ	*p* < 0.000	*p* < 0.000	*p* < 0.000	*p* < 0.000	*p* < 0.000	*p* < 0.000	*p* < 0.000	*p* < 0.000	*p* < 0.000	*p* < 0.000	*p* < 0.000
		embalm	embalm	embalm	embalm	embalm			embalm	embalm	embalm	embalm
	$1\%\;s^{-1}$ long	*p* < 0.000	*p* < 0.000	*p* < 0.000	*p* < 0.000	*p* = 0.001			*p* < 0.000	*p* < 0.000	*p* < 0.000	*p* < 0.000
		embalm	embalm	embalm	embalm	embalm			embalm	embalm	embalm	embalm
	$10\%\;s^{-1}$ long	*p* < 0.000	*p* < 0.000	*p* < 0.000	*p* < 0.000	*p* = 0.001			*p* < 0.000	*p* < 0.000	*p* < 0.000	*p* < 0.000
		embalm	embalm	embalm	embalm	embalm	fresh		embalm	embalm	embalm	embalm
	$1\%\;s^{-1}$ circ	*p* < 0.000	*p* < 0.000	*p* < 0.000	*p* < 0.000	*p* < 0.000	*p* = 0.007		*p* < 0.000	*p* < 0.000	*p* < 0.000	*p* < 0.000
		embalm	embalm					embalm	embalm	embalm	embalm	
	$10\%\;s^{-1}$ circ	*p* < 0.000	*p* < 0.000					*p* = 0.001	*p* < 0.000	*p* = 0.008	*p* < 0.000	
